# Cytotoxic T Lymphocyte Trafficking and Survival in an Augmented Fibrin Matrix Carrier

**DOI:** 10.1371/journal.pone.0034652

**Published:** 2012-04-04

**Authors:** Zhaoxia Zou, Erin Denny, Christine E. Brown, Michael C. Jensen, Gang Li, Tatsuhiro Fujii, Josh Neman, Rahul Jandial, Mike Chen

**Affiliations:** 1 Division of Neurosurgery, City of Hope National Medical Center, Duarte, California, United States of America; 2 Department of Cancer Immunotherapeutics & Tumor Immunology, City of Hope National Medical Center, Duarte, California, United States of America; University of Leuven, Rega Institute, Belgium

## Abstract

Cell-based therapies have intriguing potential for the treatment of a variety of neurological disorders. One such example is genetically engineered cytotoxic T lymphocytes (CTLs) that are being investigated in brain tumor clinical trials. The development of methods for CTL delivery is critical to their use in the laboratory and clinical setting. In our study, we determined whether CTLs can migrate through fibrin matrices and if their migration, survival, and function could be modulated by adding chemokines to the matrix. Our results indicated that CTLs can freely migrate through fibrin matrices. As expected, the addition of the monocyte chemotactic protein-1 (MCP-1), also known as chemokine C-C motif ligand 2 (CCL2), to the surrounding media increased egress of the CTLs out of the fibrin clot. Interleukin (IL) -2 and/or IL-15 embedded in the matrix enhanced T cell survival and further promoted T cell migration. The interleukin-13 receptor alpha 2 specific (IL-13R alpha2) T cells that traveled out of the fibrin clot retained the capacity to kill U251 glioma cells. In summary, CTLs can survive and migrate robustly in fibrin matrices. These processes can be influenced by modification of matrix constituents. We conclude that fibrin matrices may be suitable T cell carriers and can be used to facilitate understanding of T cell interaction with the surrounding microenvironment.

## Introduction

Adoptive immunotherapy using genetically engineered cytotoxic T lymphocytes (CTLs) is being investigated as a potential treatment for cancer in the central nervous system and elsewhere [1], [2]. Pilot clinical trials have been initiated to evaluate the feasibility and safety of local-regional delivery of autologous IL13-zetakine redirected CTL clones in patients with recurrent glioblastoma (GBM). These results indicate the adoptive transfer of tumor-specific cytotoxic T cells to the tumor bed is a promising strategy, though its clinical application may be limited due to the shortcomings of previous and current delivery methods.

While systemic delivery is appealing for its convenience, non-specific targeting, high cell dosage requirements, and the presence of the blood-brain barrier are formidable obstacles. In clinical trials local delivery can be achieved using catheter-based delivery systems. Catheter-based delivery systems, however, are associated with risks such as infection and hemorrhage. Additionally, catheter-based delivery systems used at our institution present significant logistical challenges. First, injection results in the formation of a pocket of T cells at the catheter tip because cell sized particles cannot flow through the extracellular space. With point source distribution, CTLs may have to seek and find distant targets in the brain diminishing the chances of successful therapy. Second, to attain an adequate dose, multiple injections are needed. These multiple injections lengthen hospital stay creating more cost, risk, and patient inconvenience. We hypothesized that T cells could be delivered more efficiently by embedded them in a fibrin matrix placed in the resection cavity during surgery. The fibrin matrix is a commercially available (Baxter, Tisseel) biodegradable adhesive obtained by a combination of human-derived fibrinogen and thrombin, duplicating the last step of the coagulation cascade. Tisseel is normally used in neurosurgery as a dural sealant and a hemostatic agent. Studies have shown that the application of fibrin glue to the brain is safe and well tolerated [3], [4], [5].

Our approach of using fibrin glue as a vehicle for CTLs has not been previously described, though fibrin glue has been widely used for delivery of a variety of cells [6], [7], [8], [9], [10], [11], [12], [13], [14], [15]. Fibrin matrices are employed not only as a cell carrier, but also as suitable vectors for drugs and exogenous growth factors. Therefore, there exists the potential to combine cell-based therapies with modulating biological factors in the fibrin glue.

A prerequisite for use of fibrin matrices as a T cell delivery vehicle is that the cells must be able to migrate out of the fibrin clot. Additionally, these cells must survive the passage and remain efficacious tumor killers. In this study, we demonstrated the ability of T cells to robustly migrate through a bio-enhanced matrix while maintaining potency against glioma cells.

## Materials and Methods

### Reagents

Recombinant human interleukin (IL) -2 was purchased from Cell signaling. Recombinant human IL-15 and monocyte chemotactic protein-1 (MCP-1) were purchased from R&D Systems (Minneapolis, MN). Each cytokine and chemokine was reconstituted in PBS with 0.1% human serum albumin (PBS–HSA) and stored at −20°C until time of use. On the day of experimentation, aliquots were diluted in RPMI media to the final concentrations as indicated.

### Cell lines and Cultures

In our experiments, firefly luciferase (ffl+) expressing interleukin-13 receptor alpha 2 specific (IL-13R alpha2) CTLs cells were cultured in RPMI 1640 (Invitrogen) with 10% heat-inactivated fetal calf serum (FCS), 25 mmol/L HEPES, and 2 mmol/L L-glutamine supplemented with 50 U/mL rhIL-2 (Chiron). U251 glioblastoma cells were grown in Dulbecco's modified Eagle's medium supplemented with 10% heat-inactivated FCS, 25 mmol/L HEPES, and 2 mmol/L L-glutamine.

For conditioned medium, U251 cell lines were grown to 60–90% confluency and cells were then transferred to serum-free medium. 24 and 48 hours after the transfer, the cell-free supernatants were collected.

### Fibrin Matrix (Tisseel) Formulation and Dissolution

The sealer protein component (fibrinogen-containing component) was reconstituted in aprotinin solution (Baxter Healthcare), and the thrombin component was reconstituted in 30 mM CaCl_2_. Both solutions were diluted to the appropriate concentration using Trisbuffered saline (TBS) and 30 mM CaCl_2_ in TBS for the sealer protein and thrombin components, respectively. Formulation 1 was composed of 2.5 mg/ml fibrinogen and 2.5 U/ml thrombin. Formulation 2 was composed of 5 mg/ml fibrinogen and 5 U/ml thrombin. Finally, Formulation 3 was composed of 5 mg/ml fibrinogen and 1 U/ml thrombin. In select experiments tissue plasminogen activator (tPA) was used to dissolve the fibrin clots to determine the number of remaining T cells. The CellTiter-Glo Luminescent Cell Viability Assay (Promega) was performed as a surrogate for cell count.

### Incorporation of T Cells in Fibrin Matrix and Chemotaxis Assays

T cell/fibrin clot constructs were prepared in the following manner. Following harvest with trypsin–EDTA, 100 µl of ffl+ IL-13 zetakine CTLs (7.5*10^6^ cells/mL) were mixed with 150 µl sealer protein solution. 150 µl of thrombin solution was subsequently added to the sealer protein–cell solution and mixed by tapping and tilting the plates. Then 400 µl of the combined mixture was placed in 24-well 5 µm QCM Chemotaxis Cell Migration Assay plates (Millipore). Plates were incubated in a 5% CO_2_ incubator for 8, 24, or 48 hours. At each time point, the number of T cells that migrated out of the clots from the upper chamber to the lower chamber was assayed using the CellTiter-Glo Luminescent Cell Viability kit and measured using a luminescence plate reader. The number of T cells that had migrated out of the fibrin matrix was calculated according to the standard curve; then the percent migration was calculated by dividing the number of collected T cells by number of initial T cells placed in the clot and multiplying the result by 100. Cytotoxicity of IL-13 zetakine CTLs passage through the fibrin matrix may adversely affect the ability of CTLs to kill tumor cells. The potency of the CTLs was assayed in the following manner. Effector cells were used 13 to 15 days after stimulation with muromonab-CD3 (OKT3) and irradiated feeder cells. 7.5×10^5^ IL-13 zetakine CTLs per fibrin clot were plated into the upper chamber of 24 well chemotaxis assay plates. The CTLs that had migrated out were collected after 24 hours. U251 ffl+ target cells (200,000 cells/ml) were co-cultured with increasing numbers of CD8+ effector zetakine CTLs in a 5% CO_2_ incubator. After 4 or 24 hours of incubation, Dual-Glo Luciferase Assay Reagent was added to each well of the 96-well plate. The plate was incubated for 10 minutes at room temperature, and luminescence was read from 8 mm above the bottom of the plate on the luminescence plate reader.

### Human Chemokine Levels in Glioma-Conditioned Medium

Chemokine levels of MCP-1, C-C motif ligand 5 (CCL5), and C-X-C motif ligand 10 (CXCL10) were determined using their respective Human ELISA Kits (Invitrogen) as per the manufacturers' instructions. Briefly, samples, controls, MCP-1, CCL5 (data not shown), or CXCL10 standards were incubated for 2 hours at room temperature with a biotinylated Hu MCP-1, CCL5, or CXCL10 Biotin Conjugate solution, and then incubated for 30 minutes at room temperature with Streptavidin-HRP Working Solution. Absorbance detection of captured cytokines was quantified by Optical Density using a Microplate Reader.

### Stimulation of T Cells in the Fibrin Clots and Effect on Survival and Migration

IL-2 and/or IL-15 were added to the fibrin matrix to investigate whether T cell survival and migration could be enhanced. T cell–fibrin clots were prepared as described above. IL-2 (500 u/ml), IL-15 (100 ng/ml), or IL-2 + Il-15 were mixed into the fibrin clots. The IL-2 and IL-15 concentrations were chosen based on previous studies and our preliminary experiments [16]. The CellTiter-Glo ATP assay was performed to quantify CTL migration after 24 hours.

### Statistics

The means and standard deviations of the obtained data were calculated. One-way analysis of variance (ANOVA) with post-hoc testing was used for comparing means of three or more variables. Statistical analysis was carried out using SAS software (SPSS version 12.0.1) on a Microsoft Windows XP system. The threshold for statistical significance was set at 0.05.

## Results

### T Cell Migration Through Low Viscosity Fibrin Matrices

It is essential that CTLs are able to travel through the fibrin clot to reach their target. We examined the effect of viscosity on T cell migration using 6 formulations of varying thrombin and fibrinogen concentrations. T cells that had exited to the surrounding media were collected and quantified using an ATP assay as a surrogate of cell count. The CellTiter-Glo ATP assay showed a highly linear relationship between cell count and luminescence (R^2^ = .98; data not shown).

**Figure 1:**
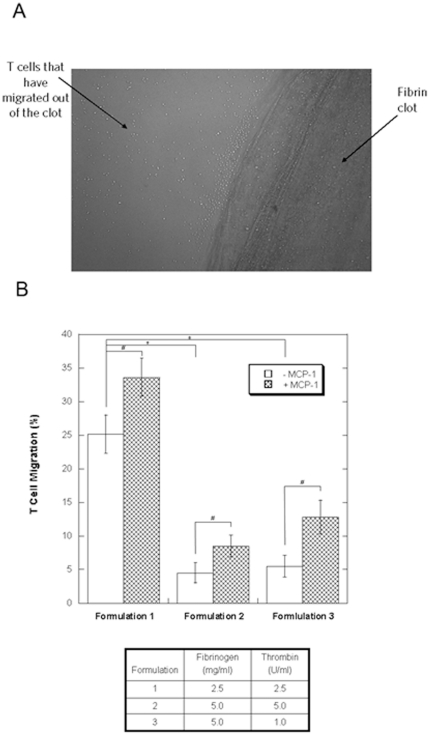
Effects of fibrin clot formulation and MCP-1 on T cell migration. (A) Microscopic evaluation (10×) of T cell migration out of fibrin matrix. 7.5×10^5^ T cells were mixed into a fibrin clot (Formulation 1). Substantial egress of T cells into surrounding media was visualized at 48 hours. (B) Effects of formulation and MCP-1 on CTL migration. CTLs were added to Tisseel clots with varying concentrations of thrombin and fibrinogen (Formulation 1–3). After 48 hours, ATP assays were performed to quantify the amount of CTLs able to migrate out of the clots and into the surrounding media. The matrix with the lowest viscosity (Formulation 1) allowed for the most T cell migration (* p<0.01). In addition to viscosity, effects of MCP-1 on migration were examined. For all formulations, the addition of MCP-1 to the surrounding media significantly increased T cell migration out of the clot (# p<0.01). The data represents the means ± SD of three independent experiments.

Formulation 1 of lower fibrinogen and thrombin concentrations was not only less viscous but also allowed the most T cells (25%) to move out of the fibrin glue (p<0.01) (Figure 1). Formulation 2 (5.0 mg/ml fibrinogen and 5.0 U/ml thrombin) and Formulation 3 (5.0 mg/ml and 1.0 U/ml) allowed only 5% of T cells to migrate through the clot. T cells were not capable of passaging through the fibrin matrix when concentrations of fibrinogen and thrombin exceeded 5.0 mg/ml and 1.0 U/ml respectively (data not shown). At concentrations lower than those reported in Formulations 1, the fibrin glue did not form a clot, and therefore these formulations were not further analyzed.

### Chemoattractants in the Surrounding Media Increase CTL Migration Out of the Fibrin Clot

MCP-1 is a known T cell chemokine; its ability to penetrate into fibrin matrices, however, was unknown. We indirectly examined if MCP-1 could diffuse into the fibrin clot to promote CTL trafficking. As shown in Figure 1, the addition of MCP-1 to the media outside of the clot significantly increased CTL migration in the three formulations shown (p<0.01).

**Figure 2:**
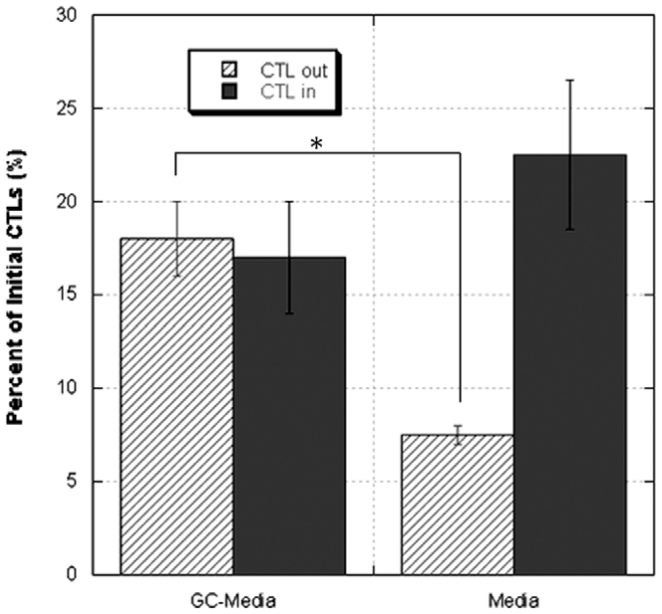
Effect of media on T cell migration and quantification of viable T cells remaining in the fibrin matrix. CTLs were mixed with Tisseel (Formulation 1) and cultured in either glioma-conditioned media (GC-Media) or standard culture media (Media). Quantification of cells performed 48 hours after incubation showed GC-Media to significantly increase T cell migration out of the fibrin matrix (* p<0.05). The combined total of cells remaining in and migrating outside the clot (reported as a percentage of the initial cell number) was approximately the same (∼30%) regardless of the type of media used. The data represents the means ± SD of three independent experiments.

In addition to secreting other CTL chemokines such as IL-8, tumors can regulate activation of normal T cell expression and secretion [17]. The collective effect of these chemokines on T cell trafficking from the fibrin clot was assessed (Figure 2). CTLs in the fibrin matrices were exposed to standard culture media or conditioned media obtained from culturing U251 glioblastoma cells. There was significantly (p<0.05) more migration out of the clot when glioma-conditioned media was used.

The fate of T cells remaining in the clot was also assessed because it was unclear if those cells were trapped or had perished. The clots were dissolved with tPA, and the cells were quantified using an ATP assay (Figure 2). Regardless of the type of media, the total number of cells—CTLs in or out of the clot—was slightly over 30% of the initial cell count. This indicated that one limiting factor was T cell demise within the fibrin matrix.

### Chemokine Level in Glioma-Conditioned Medium

**Figure 3:**
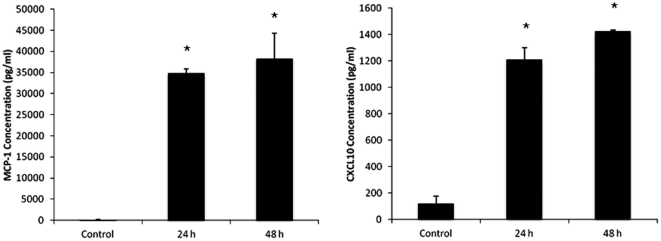
Cytokine concentrations in glioma-conditioned media (GC-Media) and standard culture media (Media). Cytokine concentrations were measured using Human ELISA Kits. (A) Increased MCP-1 concentrations in GC-Media (* p<0.0001, 24 h or 48 h vs. control). (B) Increased CXCL10 concentrations in GC-Media (* p<0.0001, 24 h or 48 h vs. control). The data represents the means ± SD of three independent experiments.

We demonstrated that there was significantly more migration out of the clot when glioma-conditioned media was used compared to standard culture media. To further investigate the mechanism for the observed trafficking of T cells in glioma-conditioned media, we measured chemokine levels. MCP-1 and CXCL10 were detected at high levels in glioma-conditioned supernatants compared to standard culture media (p<0.01) (Figure 3). Thus, the observed migrational response in Figure 2 may be a consequence of chemokine-dependent directional migration.

### IL-2 and IL-15 Alone or in Combination Enhances T Cell Survival and Promotes Trafficking

**Figure 4:**
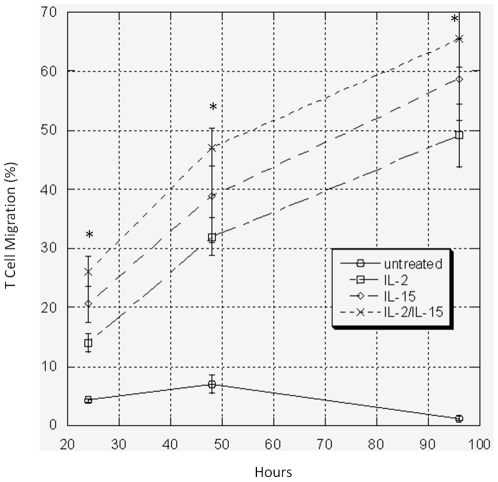
Effects of IL-2 and IL-15 on CTL migration. IL-2 (500 u/ml), IL-15 (100 ng/ml), or the combination of IL-2/IL-15 was added into the fibrin clots (Formulation 1). ATP assays were performed to quantify the number of T cells that had migrated out of the clots after 24, 48, and 96 hours. T cell migration significantly increased out of treated clots containing IL-2, IL-15, or both compared to untreated clots. The data represents the means ± SD of three independent experiments (* p<0.01 IL-2, IL-15, or IL-2/IL-15 vs. untreated).

Cell survival after implantation is a major concern. To enhance survival and potentiate trafficking, the cytokines IL-2 and IL-15, alone or in combination, were added to the fibrin matrix. Prior to this experiment, we confirmed that IL-2 and IL-15 caused expansion and an increase in the diameter of CTLs, the later reflective of T cell health (data not shown). Addition of IL-2 (500 u/ml), IL-15 (100 ng/ml), or IL-2+Il-15 into the fibrin clot dramatically (p<0.01) increased T cell egression at multiple time points (Figure 4). In contrast, the CTL migration in the untreated group decreased to almost 0% after 96 hours. The most trafficking was seen when IL-2 and IL-15 were combined; 65% of CTLs had migrated out. These findings suggest the proliferative effect of the cytokines is preserved in the fibrin matrix.

### IL-13R alpha2 CTLs That Migrate Out of the Matrix Remain Potent Against U251 Glioma Cells

**Figure 5:**
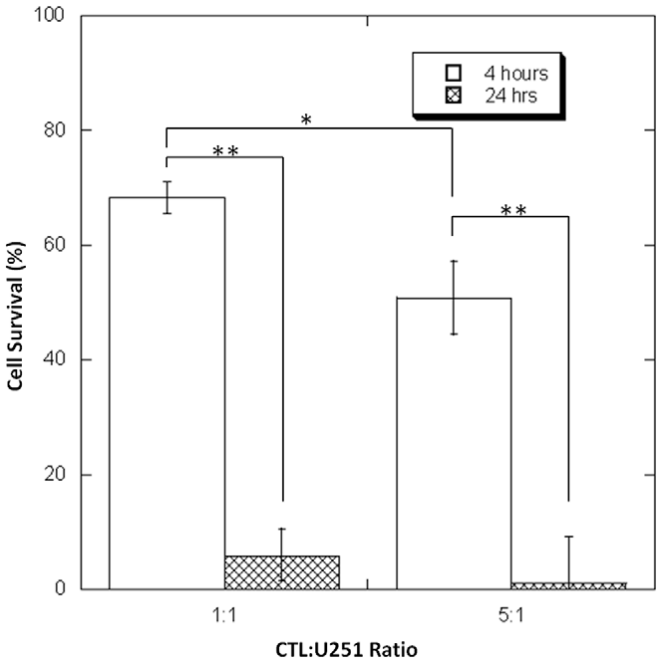
Cytotoxicity of post-migration IL-13 zetakine CTLs. Post-migration IL-13 zetakines were co-cultured with U251 glioma cells at a ratio (CTL∶U251) of 1∶1 or 5∶1. The viability of U251 cells was determined using the Dual-Glo Luciferase Assay after 4 hours (A) and 24 hours (B) of incubation time. Increasing the co-culture ratio to 5∶1 significantly decreased glioma cell survivability (* p<0.01). Regardless of the co-culture ratio, glioma cells died in a time-dependent manner (** p<0.001). The data represents the means ± SD of three independent experiments.

Whether CTLs are still potent after migrating out of the fibrin clot is unknown. U251 glioma cells expressing firefly luciferase (U251 ffl+) were co-cultured with increasing numbers of glioma-specific primary IL-13 zetakine CTLs that had migrated out of the fibrin matrix (Figure 5). Although U251 cells died in a time dependent manner, glioma cell death was dose-dependent (in terms of CTL to U251 ratio) only at a shorter treatment time (4 hours). 24 hours after incubation U251 cells were almost completely eradicated when cultured with a five fold greater number of CTLs.

## Discussion

Adoptive immunotherapy is a theoretically elegant approach for the treatment of brain tumors; however, the optimal method to deliver T cells has not been developed. We hypothesized that in lieu of the catheter-based delivery system, T cells can be distributed by using a fibrin matrix carrier that is placed into the resection cavity at the time of surgery. This method would confer potential advantages. One is that the viscous nature of the substance improves localization of the T cells to the tumor bed, preventing washout into the subarachnoid space. Another potential advantage is that catheter implantation is not required and therefore certain risks are avoided. The most important potential benefits relate to dose and distribution. A much larger dose can be delivered. Additionally, bioactive reagents can be added to the fibrin matrix to enhance the survival and function of the T cells.

Our finding that CTLs can passage through low viscosity fibrin matrices is consistent with studies reporting migration through Tisseel of fibroblasts, chondrocytes, neutrophils and monocytes [18], [19], [20], [21]. The concentration of fibrinogen and thrombin that we determined as optimal (2.5 mg/ml fibrinogen and 2.5 U/ml thrombin) is similar to those reported in Cox et al.'s study which examined fibroblast migration through fibrin clots (5–17 mg/mL fibrinogen and 1–167 U/mL thrombin).

Though it was obvious that high concentrations of fibrinogen were inhibitory, the active or passive mechanism(s) responsible for CTL egress out of Tisseel have not been defined. In vivo, tumor cells secrete chemoattractants such as MCP-1 which play an important role in the host antitumor immune response by recruiting CD4+ and CD8+ cells [22], [23], [24]. It was uncertain if CTL chemotactic factors could diffuse from the surrounding media into the clot to promote migration. We have found that glioma cell line U251 produces sufficient levels of MCP-1 to chemoattract T cell migration through fibrin clots in vitro. Our data indicated that MCP-1 containing or glioma-conditioned media—the later theoretically full of MCP-1 and other chemokines—significantly enhanced T cell migration suggesting that targeting of remote tumor cells is possible.

We allow that factors other than MCP-1 can enhance T cell migration. Although no other study has examined CTL migration using a fibrin clot, Nishimura et al. have shown that chemokines such as CXCL10, also referred to as interferon gamma-induced protein 10 (IP-10), can play a critical role in the recruitment of Tc1 effector cells to the brain tumor site [25]. Our present study showed that CXCL10, in fact, was present in high concentrations in glioma-conditioned media and could potentially promote CTL migration out of the fibrin clot. In addition to the identity of chemokines, other variables such as the amount or type of chemokine-secreting residual tumor may be important.

Even under optimal circumstances, without additional aid only about one-fourth of the CTLs were able to leave the fibrin matrix. We observed that most had perished by days 3–6. Survival varied depending on the initial health of the T cells which corresponded to their status in the stimulation cycle. Of the T cells remaining in the matrix, over two thirds of the initial cells were no longer viable. Improving viability would be expected to increase migration due to an enlarged pool of available cells.

Adoptively transferred antigen-specific CTLs are highly dependent on exogenous cytokines such as IL-2, IL-7, IL-15 and IL-21 for their continued growth and survival [26]. Systemic administration of IL-2 has been used to enhance T cell expansion and persistence in vivo [27]. Administration of lowdose IL-15 has been shown to promote the persistence of adoptively transferred tumor-specific T cells in murine tumor models; however, the systemic toxicity and the expansion of unwanted cells, including regulatory T cells, limit the clinical value of this strategy [28], [29]. We demonstrated that IL-2 and IL-15 added to the fibrin clot improved survival, possibly caused proliferation, and subsequently increased T cell migration by 2–3 fold.

Migration to the target is insufficient if the T cells lose their capacity to kill the tumor cells during passage. IL-13 zetakine CTLs have been previously shown to recognize and kill glioblastomas and medulloblastomas expressing IL-13R alpha2 [30], [31]. In fact, IL-13 zetakine CTLs have been shown to recognize and eliminate brain tumor stem-like initiating cells [32]. Our data indicated that IL-13 zetakine CTLs that had moved out of the fibrin matrix killed U251 glioma cells in a time and dose dependent fashion. The optimal dose required in vivo has yet to be determined. However, the observed dose dependency suggests that larger doses possible with fibrin matrix delivery would confer a significant advantage.

We have shown that robust migration can be achieved with IL-2 and IL-15, chemotaxis mechanisms are not impaired by the matrix, and that CTLs that have passaged through the matrix remain potent. The significant logistical and biological advantages associated with this technique are promising but will need to be confirmed in vivo and in future clinical trials.
